# Discrepant Amplification Results during the Development of an Assay Leads to Reclassification of Two AIDS Reagent Repository HIV-2 Isolates as HIV-1

**DOI:** 10.1371/journal.pone.0096554

**Published:** 2014-05-05

**Authors:** Linda L. Jagodzinski, Ying Liu, Holly R. Hack, Catherine Kibirige, Sheila A. Peel, Mark M. Manak

**Affiliations:** 1 U.S. Military HIV Research Program, Walter Reed Army Institute of Research, Silver Spring, Maryland, United States of America; 2 U.S. Military HIV Research Program, Henry M. Jackson Foundation for the Advancement of Military Medicine, Silver Spring, Maryland, United States of America; University of Pittsburgh, United States of America

## Abstract

The development and verification of HIV-2 assays depends in part on the availability of well-characterized samples, including those from reagent repositories. During the development of an HIV-2 RNA quantification assay, two HIV-2 viral isolates (CDC 301340 and CDC 301342) obtained from the NIAID AIDS Reagent and Reference Repository were not detected leading to an investigation. Two HIV-2 primers/probe sets of known performance in real-time viral RNA quantification assays, targeting different regions of the virus, also failed to generate RT-PCR products for these two isolates. These isolates were tested in the HIV-1 specific COBAS AmpliPrep/COBAS TaqMan HIV-1 Test v2.0 (Roche Molecular Diagnostics) and were quantified at high copy number. Other HIV-2 isolates tested were not amplified in the COBAS HIV-1 TaqMan assay. Furthermore, the discrepant isolates were highly reactive in an HIV-1 p24 antigen test while the other HIV-2 isolates showed very weak, if any, cross-reactivity with the HIV-1 p24 assay. Phylogenetic tree analysis of sequences from the protease-reverse transcriptase regions of the discrepant HIV-2 isolates mapped with HIV-1 Group M, Subtype CRF02_AG confirming these isolates were of HIV-1 origin and had been misclassified as HIV-2. The use of misclassified isolates in the verification of molecular and immunological assays can lead to misinterpretation of test results, misdirection of efforts into assay redesign and increased development costs. The results of this study were shared with the NIAID AIDS Reagent Program, leading to the reclassification of the two discrepant isolates as HIV-1.

## Introduction

Infection with Human Immunodeficiency Virus Type 2 (HIV-2), as in the case of HIV-1, is associated with opportunistic infections and AIDS. HIV-2 infections are mainly confined to regions of West Africa where an estimated 1-2 million people are infected [1], [2]. However, infections have also been identified in Europe, Asia, the Caribbean and the United States [3], [4], [5]. The virus is characterized by a high degree of genetic diversity and has been classified into at least eight groups, A and B being the most common [6], [7]. Serological diagnosis and differentiation of HIV-2 infection is difficult due to cross reactivity with HIV-1 antibodies [8]. The BioRad Multispot HIV-1/HIV-2 Rapid Test is the only U.S. Food and Drug Administration (FDA) test cleared for detection/differentiation of HIV-1 and HIV-2 antibodies [2]. The development of commercial HIV-2 plasma RNA assays has been hampered by low HIV-2 prevalence, slower progression to AIDS, lower viral load set points in HIV-2 infected individuals (as compared to HIV-1) and limited sequence data from well-characterized isolates. The availability of highly sensitive and specific HIV-2 RNA assays would be useful to confirm or rule-out HIV-2, assist in therapeutic management of infection, or, as recommended by the proposed CDC algorithm, to confirm or rule-out HIV-1/HIV-2 dual infection [3], [9].

Several laboratory developed assays for HIV-2 RNA were developed to address the need for detection and quantification of HIV-2 nucleic acid in plasma, however, standardization, validation and regulatory approvals have been challenging [8], [10], [11], [12], [13], [14]. A significant challenge in HIV-2 assay validation is the limited resources for diverse HIV-2 isolates for nucleic acid source material. HIV-2 viral culture stocks are the only readily available source of well-characterized material. This study reports an investigation of discrepant results obtained in reverse transcription HIV-2 quantitative real-time PCR assays (RT-qPCR) for two HIV-2 viral stocks received from the NIAID AIDS Reagent and Reference Repository.

## Materials and Methods

### Viral Stocks and EDTA Plasma Diluent

Twelve HIV-2 viral stocks were obtained from the NIAID AIDS Reagent and Reference Repository (10 group A, 1 group B and 1 unknown). Additional virus culture isolates tested included the HIV-2 group B isolate (PB0012902206) obtained from SeraCare, Inc. (Gaithersburg, MD). An HIV-1 isolate (91US_4) obtained from the Humoral Immunology Laboratory of the U.S. Military HIV-1 Research Program (Drs. Bruce Brown and Victoria Polonis) was used as a virus specificity control. Prior to testing, all viral stocks were diluted one thousand fold in EDTA plasma (Biological Specialty Corporation, Comar, PA).

### HIV-2 Quantitative RT-qPCR Assays

#### HIV-2 Calibrator/Standard

An HIV-2 viral stock (NIH-Z, group A) was purchased from Advance BioTechnologies, Inc. (Columbia, MD) for use as a quantification standard in real-time RT-qPCR assays (7.2 E+10 HIV-2 copies/ml by electron microscopy particle count). The NIH-Z viral stock was diluted in normal human EDTA plasma to obtain a working stock of 7.2E+09 HIV-2 copies/ml. Ten-fold serial dilutions were performed in EDTA plasma to generate an HIV-2 panel of quantification standards with concentrations ranging from 7.2 E+06 to 7.2 E+01 copies/ml.

#### HIV-2 Primers and Probe Sets

Two HIV-2 primers/probe sets of known or published performance were selected to confirm discordant RT-qPCR test results. The HIV-2 primers/probe set designated as SM, targeting the LTR region as defined in Delarue et al [12], was selected based upon equivalent amplification of HIV-2 groups A and B. SM primers and probe were synthesized by Integrated DNA Technologies (Coralville, IA). A second primers/probe set designated as PD targeting the HIV-2 *gag* gene was selected for use in detecting and quantifying an alternative region of the viral genome. The PD set is included in the Advance Real-Time PCR HIV-2 Detection Kit from Primer Design LTD (South Hampton, UK). The product insert claims equivalent quantification of HIV-2 groups A and B.

#### RNA Extraction and Amplification

HIV-2 viral RNA was extracted from 0.2 ml of diluted virus or NIH-Z standards and eluted in 50 µl of kit diluent using the MinElute Viral Extraction Kit (QIAGEN, Valencia, CA). Amplification reactions were performed at 25 µl and contained 10 µl of purified RNA, 0.6 µM forward and reverse primers, 0.2 µM probe and amplification reagents according to kit insert (Superscript III Platinum One-Step Quantitative RT-PCR system: Life Technologies, Carlsbad, CA). Amplification was performed using a 7500 Fast Dx Real-Time PCR instrument (Life Technologies, Carlsbad, CA). HIV-2 RNA amplification parameters were the same for all RT-qPCR assays; 1) reverse transcription at 50°C for 30 minutes, 2) activation of the DNA polymerase at 95°C for 2 minutes, 3) 5 cycles of amplification at 95°C for 15 seconds, 52°C for 10 seconds and 60°C for 1 minute and 4) 40 cycles of amplification at 95°C for 15 seconds, 57°C for 10 seconds and 60°C for 1 minute with fluorescent read. HIV-2 viral concentrations were extrapolated from the NIH-Z standard curve.

#### Commercial Test Kits

A subset of viral isolates, including the discrepant samples, was also tested in HIV-1 p24 Antigen and RNA assays. The HIV-1 p24 Antigen assay from Perkin Elmer (Waltham, MA) and the Roche COBAS AmpliPrep/COBAS TaqMan HIV-1 Test v2.0 (Roche Diagnostics, Indianapolis, IN), an FDA cleared HIV-1 RNA test that does not detect HIV-2, were performed following the manufacturer's recommendations. A 918 base pair sequence from the HIV-1 polymerase gene was generated for each discrepant isolate using the TRUGENE HIV-1 Genotyping Test (Siemens Healthcare Diagnostics, Valencia, CA).

## Results

**Figure 1:**
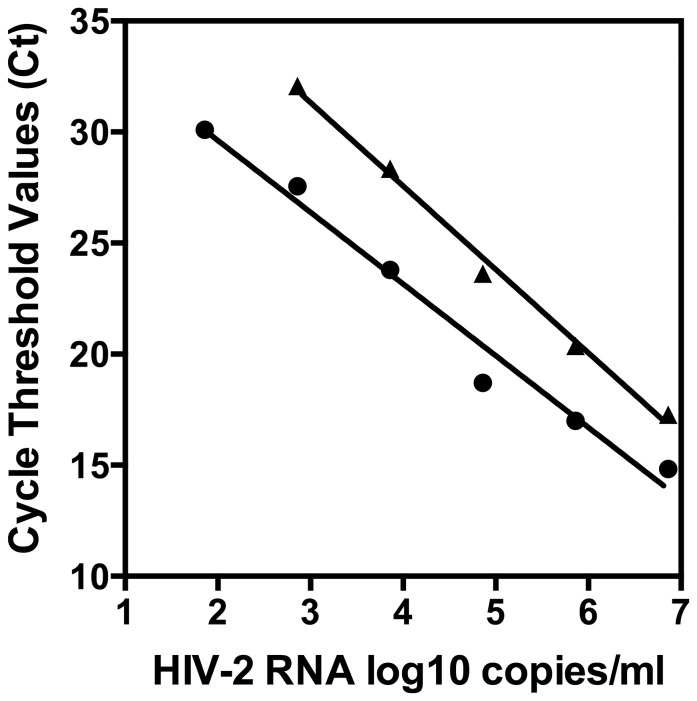
Performance of two HIV-2 primers/probe sets in real-time RT-qPCR assays. Two HIV-2 primers/probe sets were assessed for amplification of serial dilutions of an HIV-2 isolate, NIH-Z, under standard ampification conditions that were not optimized for each primers/probe set. Primers/probe sets, designated as PD  =  Primer Design LTD HIV-2 PCR Kit and SM  =  Delarue et al [12], showed linear amplification profiles that were parallel. PD  =  •. SM  =  ▴.

Two HIV-2 primers/probe sets developed for use in quantification of HIV-2 RNA, one by Delarue et al 2013 (SM) and a commercially available set from Primer Design (PD) were selected for amplification of HIV-2 RNA in real-time RT-qPCR assays. Although optimization was not performed, both primers/probe sets performed reasonably well using standard cycling parameters and amplification reagents. Successful quantitation over a broad dynamic range from two to six log_10_ was obtained for the NIH-Z standard (Figure 1). The amplification plot showed parallel performance for both primers/probe sets (Figure 1). The PD set demonstrated lower cycle threshold values than the SM set yielding a slope of -3.232 indicating good amplification efficiency and a R^2^ value of 0.9774. The SM primers/probe set had a slope of -3.758 indicating a lower efficiency of amplification and a R^2^ value of 0.997. The lower amplification efficiency of the SM primers/probe set resulted in no amplification of the HIV-2 NIH-Z standard at the lowest HIV-2 concentration. The PD primers/probe set provided higher HIV-2 viral RNA concentrations for the HIV-2 viral isolates tested than the SM primers/probe set (Table 1) which is most likely due to the decreased amplification efficiency of the SM set. Further optimization and validation of these primers/probe sets would be required to allow for reliable quantification of absolute copy number. However, based upon linearity and quantitation relative to the NIH-Z standard, these primers/probe sets showed that the HIV-2 viral stocks had high titers, indicating that these stocks were suitable for use in assessing the performance of laboratory developed assays.

**Table 1:** Performance of HIV-1/2 viral stocks in real-time RT-qPCR and HIV-1 p24 antigen tests.

HIV Viral Stock Information				HIV-2 Tests		HIV-1 Tests	
NIAID	Isolate	Country	HIV-1/2	PD	SM	Roche TaqMan	Perkin -Elmer
Catalog #	Name	of Origin	Subtype/Group	RNA copies/ml (log10)	RNA copies/ml (log10)	RNA copies/ml (log10)	p24 pg/ml
N/A	91US_4	USA	1B	TND	TND	5.12	236.0
N/A	PB0012902206	Cote d'Ivoire	2B	5.58	3.92	TND	0.2
3930	CDC 310319	Cote d'Ivoire	2B	5.60	4.02	TND	0.5
3932	CDC 77618	Cote d'Ivoire	2A	6.68	5.22	TND	0.0
3931	CDC 310340	Cote d'Ivoire	2A	TND	TND	5.34	230.0
3652	CDC 310342	Cote d'Ivoire	2 UNK	TND	TND	5.22	246.0
599	CBL-23	Gambia	2A	6.72	5.09	–	–
600	CBL-20	Gambia	2A	6.46	5.44	–	–
803	MVP-15132	Senegal	2A	6.58	5.17	–	–
1337	D194	Gambia	2A	6.13	4.90	–	–
3512	7924A	Cote d'Ivoire	2A	6.06	2.82	–	–
3513	60415K	Senegal	2A	5.81	4.99	–	–
3648	CDC 310248	Cote d'Ivoire	2A	4.83	4.00	–	–
3649	CDC 310072	Cote d'Ivoire	2A	6.33	5.19	–	–

Individual viral stocks identified as HIV-2 were obtained from the NIAID AIDS Reagent and Reference Repository and SeraCare, Inc. The HIV-1 subtype and HIV-2 group identification is based upon data sheets provided by NIAID and from publications. An HIV-1 Subtype B isolate from the United States (91US_4) was used as an HIV-1 control. Viral RNA was extracted and tested in HIV-2 real-time RT-qPCR assays. Viral isolates selected for testing in the Roche COBAS AmpliPrep/COBAS TaqMan HIV-1 v2.0 quantitative RNA assay and the Perkin-Elmer p24 Antigen test included the two viral isolates that were not amplified in the HIV-2 real-time RT-qPCR assays, three HIV-2 viral isolates that amplified well and the HIV-1 91US_4 isolate. Although the HIV-2 RT-qPCR assays were not optimized, the HIV-2 amplifications were robust for the majority of the isolates tested as reflected in the HIV-2 RNA concentrations reported based upon relative values extrapolated from the NIH-Z standard for each primers/probe set. The CDC 310340 and CDC 310342 viral stocks were not amplified in HIV-2 RT-qPCR assays but were quantified and reactive in HIV-1 specific tests. PD  =  Primer Design LTD HIV-2 PCR Kit primers/probe. SM  =  Delarue et al [12] primers/probe set. TND  =  Target Not Detected. UNK is unknown group. Viral isolate was not tested (−).

The PD and SM primers/probe sets demonstrated robust amplification in eleven HIV-2 viral isolates, but failed to amplify either the CDC 310340 or CDC 310342 isolates (Table 1) confirming the amplification results obtained in our laboratory developed test. Repeat testing of these two discrepant HIV-2 isolates at 10 and 100 times greater concentration resulted in no amplification, except for a single low-level detection (36.23 Ct) of the highest CDC 310342 concentration tested in one out of three replicates with the PD primers/probe set. To verify viral species specificity, an HIV-1 subtype B isolate (91US_4) was tested and was not amplified by the two HIV-2 primers/probe sets (Table 1).

Analysis of a subset of the HIV-2 isolates, including the discrepant isolates by COBAS AmpliPrep/COBAS TaqMan HIV-1 Test v2.0 (Roche TaqMan HIV-1 v2.0) resulted in quantification at 5.34 and 5.22 log_10_ HIV-1 RNA copies/ml for CDC 310340 and CDC 310342, respectively, while the HIV-1 91US_4 was quantified at 5.12 log_10_ HIV-1 RNA copies/ml (Table 1). No amplification was observed for the other HIV-2 isolates tested: PB0012902206, CDC 310319 and CDC 77618.

These HIV-2 supernatants were tested in the HIV-1 p24 antigen assay. High titers of HIV-1 p24 antigen were observed for the HIV-1 (91US_4) control stock as expected, but were also high for the two discrepant HIV-2 stocks, CDC 310340 and CDC 310342. HIV-1 p24 antigen reactivity was low in the other HIV-2 viral stocks tested (Table 1).

**Figure 2:**
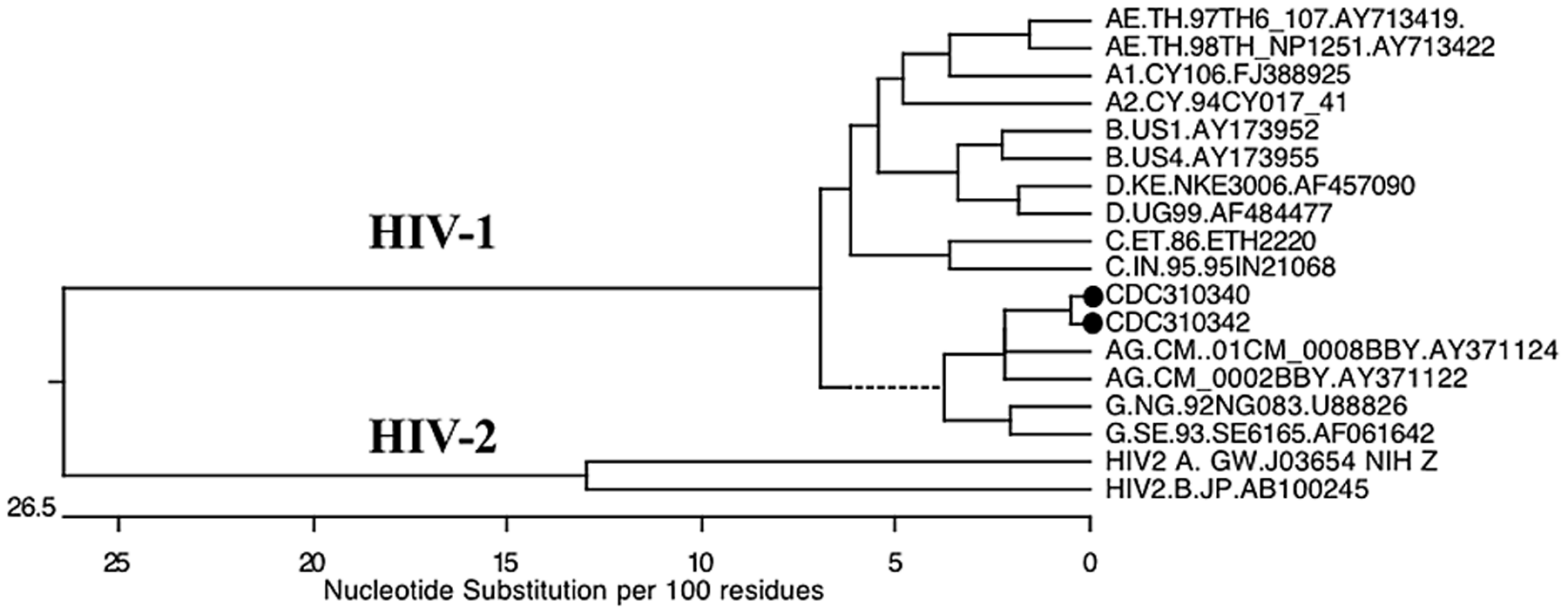
Phylogenetic Tree Analysis of Protease/Reverse Transcriptase Sequences for CDC 310340 and CDC 310342 Viral Stocks. HIV-1 sequences were obtained using the Siemens' TRUGENE HIV-1 Genotype Test, analyzed using MegAlign version 9.0.4 (DNASTAR, Inc) and subtyped against the HIV Sequence Database using BLAST (www.hiv.lanl.gov/content/sequence/BASIC_BLAST.html). Both sequences were found to align with HIV-1 Group M, subtype CRF02_AG. The homology between the two sequences was 98% with 0.3% divergence. Both sequences are designated in the tree with a •. The dotted line indicates a negative branch length, which is a result of averaging.

HIV-1 sequences for the protease/reverse transcriptase gene regions of the discrepant isolates, CDC 310342 and CDC 301342, were generated in the TRUGENE HIV-1 Genotype Test. BLAST analysis of the sequences against the HIV database (www.hiv.lanl.gov) and phylogenetic tree analysis (DNASTAR Inc.; MegAlign; Madison, WI) identified both sequences as HIV-1, subtype CRF02_AG (Figure 2). Sequences were submitted to GenBank with accession numbers KF031147 (CDC 310340) and KF031148 (CDC 310342). Sequence analysis indicates close homology between the two isolates (98%; 0.3% divergence). To rule out the possibility that these results may be due to a laboratory mix up of samples a second source vial of the two discrepant HIV-2 stocks (CDC 310340 and CDC 310342) was obtained from the NIAID AIDS Reagent and Reference Repository. The HIV PCR and sequence testing was repeated on the new vials, with comparable results. Thus, the results of these studies indicate that the two isolates are HIV-1, and not HIV-2.

## Discussion

While worldwide prevalence of HIV-2 is very low, the lack of commercial assays for accurate diagnosis, confirmation, and clinical/therapeutic management of HIV-2 infection seriously impedes care and treatment of HIV-2 infected individuals. Reliable HIV-2 reference sources and standards are critical for assay validation, platform verification, and comparative assessment of HIV-2 nucleic acid assays. In the process of developing an HIV-2 RNA qPCR assay, discrepant test results were obtained for two HIV-2 viral culture stocks received from the NIAID AIDS Reagent and Reference Repository. Comparative testing was performed using HIV-2 primers/probe sets that have been previously shown to be specific for HIV-2 RNA quantification. These comparative HIV-2 primers/probe sets showed good linearity (R^2^ values >0.97) over a broad dynamic range when evaluated on the NIH-Z standards (Figure 1). The PD and SM primers/probe sets detected and quantified HIV-2 RNA in eleven of the thirteen HIV-2 isolates tested but failed to amplify the CDC 301340 and CDC 310342 HIV-2 viral stocks, or the HIV-1 isolate. In contrast, the Roche TaqMan HIV-1 v2.0 assay, which claims specificity for HIV-1 only, detected very high viral RNA concentrations in CDC 301340 and CDC 310342 as well as the HIV-1 91US_4 isolate. Additional evidence that the two discrepant HIV-2 viral isolates were actually of HIV-1 origin is the high HIV-1 p24 antigen titers observed as compared to the other HIV-2 isolates tested. The nucleic acid sequences generated for the discrepant HIV-2 viral isolates in the TRUGENE HIV-1 Genotype Test were identified by phylogenetic tree analysis as HIV-1 Group M, subtype CRF02_AG sequences. As both isolates were obtained from Cote d′Ivoire, a homology of 98% although high, can be observed in transmission clusters [15].

The CDC 310340 and CDC 310342 viral stocks were isolated from blood donors in Cote d′Ivoire in 1997 and 1998, and several publications since have reported their use in co-receptor and infectivity studies. In one study, both of the discrepant HIV-2 isolates were omitted from a phylogenetic tree constructed from partial env sequences in the C2/V3 region for twelve new HIV-2 isolates [16]. No discussion was provided as to why the sequences for these two viral isolates along with a third were omitted. In another study, CDC 310342 was used to assess inhibition of HIV replication in human cells by Debio-025 [17]. This viral isolate was found to be sensitive to inhibition with an IC_50_ value similar to HIV-1, whereas the other HIV-2 isolate, CDC 310319, was insensitive. Six of 15 amino acid sequence within the CypA binding domains of the CA protein of CDC 310342 differed from the other HIV-2 isolates tested, but was identical to the sequence of HIV-1 subtype A. In a third paper describing a co-receptor targeted inhibitor study, CDC 310340 failed to replicate in Δ32-CCR5 PBMC and in CEMx174 cells whereas, the other four HIV-2 isolates were able to replicate in these cells [18]. The results of these studies are consistent with our analysis that CDC 310340 and CDC 310342 are not HIV-2.

The performance of CDC 310340 and CDC 310342 isolates in laboratory developed HIV-2 RT-qPCR assays were not consistent with the performance of the other eleven HIV-2 isolates tested. The lack of amplification using HIV-2 specific primers/probe sets, high titers demonstrated with the HIV-1 p24 antigen test, high HIV-1 viral load test results obtained by the Roche TaqMan HIV-1 v2.0 test, and performance in an HIV-1 specific genotype test confirm that both source vials received for each isolate were HIV-1. Contamination and mishandling of the viral stocks within the laboratory were ruled out as comparable test results were obtained on second source vials for CDC 310340 and CDC 310342. However, an earlier contamination event or dual infection with HIV-1/HIV-2 with HIV-1 out growth in culture cannot be ruled out. As assays increase in sensitivity and specificity, resource materials can benefit from reevaluation, as shown in this case, with the reclassification of two HIV-2 NIAID stocks as HIV-1 subtype CRF02_AG based on the sequence of the protease/reverse transcriptase gene regions. This report provides a cautionary note to researchers of ensuring that reagents are properly identified and classified and underscores the importance of the availability of properly classified and well-characterized reagents for use as reference material.

## References

[1] Gao F, Yue L, White AT, Pappas PG, Barchue J, et al (1992) Human infection by genetically diverse SIVSM-related HIV-2 in west Africa. Nature 358: 495–499. 1641038 10.1038/358495a0

[2] Peterson K, Jallow S, Rowland-Jones SL, de Silva TI (2011) Antiretroviral Therapy for HIV-2 Infection: Recommendations for Management in Low-Resource Settings. AIDS Res Treat 2011: 463704. 21490779 10.1155/2011/463704 PMC3065912

[3] Campbell-Yesufu OT, Gandhi RT (2011) Update on human immunodeficiency virus (HIV)-2 infection. Clin Infect Dis 52: 780–787. 21367732 10.1093/cid/ciq248 PMC3106263

[4] Centers for Disease Control and Prevention (2011) HIV-2 Infection Surveillance—United States, 1987-2009. MMWR Morb Mortal Wkly Rep 60: 985–988. 21796096

[5] Ibe S, Yokomaku Y, Shiino T, Tanaka R, Hattori J, et al (2010) HIV-2 CRF01_AB: first circulating recombinant form of HIV-2. J Acquir Immune Defic Syndr 54: 241–247. 20502347 10.1097/QAI.0b013e3181dc98c1

[6] Damond F, Apetrei C, Robertson DL, Souquiere S, Lepretre A, et al (2001) Variability of human immunodeficiency virus type 2 (hiv-2) infecting patients living in france. Virology 280: 19–30. 11162815 10.1006/viro.2000.0685

[7] Lemey P, Pybus OG, Wang B, Saksena NK, Salemi M, et al (2003) Tracing the origin and history of the HIV-2 epidemic. Proc Natl Acad Sci U S A 100: 6588–6592. 12743376 10.1073/pnas.0936469100 PMC164491

[8] Guyader M, Emerman M, Sonigo P, Clavel F, Montagnier L, et al (1987) Genome organization and transactivation of the human immunodeficiency virus type 2. Nature 326: 662–669. 3031510 10.1038/326662a0

[9] New York State Department of Health AIDS Institute (2012) Human Immunodeficiency Virus Type 2 (HIV-2). HIV Clinical Resource: New York State Department of Health AIDS Institute.

[10] Damond F, Benard A, Balotta C, Boni J, Cotten M, et al (2011) An international collaboration to standardize HIV-2 viral load assays: results from the 2009 ACHI(E)V(2E) quality control study. J Clin Microbiol 49: 3491–3497. 21813718 10.1128/JCM.02389-10 PMC3187346

[11] Berry N, Herrera C, Cranage M (2011) Detection, quantification, and characterisation of HIV/SIV. Methods Mol Biol 665: 133–160. 21116800 10.1007/978-1-60761-817-1_9

[12] Delarue S, Didier E, Damond F, Ponscarme D, Brengle-Pesce K, et al (2013) Highly sensitive plasma RNA quantification by real-time PCR in HIV-2 group A and group B infection. J Clin Virol 58: 461–467. 24008204 10.1016/j.jcv.2013.08.003

[13] Chang M, Gottlieb GS, Dragavon JA, Cherne SL, Kenney DL, et al (2012) Validation for clinical use of a novel HIV-2 plasma RNA viral load assay using the Abbott m2000 platform. J Clin Virol 55: 128–133. 22832059 10.1016/j.jcv.2012.06.024 PMC3444162

[14] Styer LM, Miller TT, Parker MM (2013) Validation and clinical use of a sensitive HIV-2 viral load assay that uses a whole virus internal control. J Clin virol 58 suppl 1e127–133. 24342472 10.1016/j.jcv.2013.09.007

[15] Hue S, Clewley JP, Cane PA, Pillay D (2004) HIV-1 pol gene variation is sufficient for reconstruction of transmissions in the era of antiretroviral therapy. AIDS 18: 719–728. 15075506 10.1097/00002030-200403260-00002

[16] Owen SM, Ellenberger D, Rayfield M, Wiktor S, Michel P, et al (1998) Genetically divergent strains of human immunodeficiency virus type 2 use multiple coreceptors for viral entry. J Virol 72: 5425–5432. 9620997 10.1128/jvi.72.7.5425-5432.1998 PMC110175

[17] Ptak RG, Fu W, Sanders-Beer BE, Dickerson JE, Pinney JW, et al (2008) Cataloguing the HIV type 1 human protein interaction network. AIDS Res Hum Retroviruses 24: 1497–1502. 19025396 10.1089/aid.2008.0113 PMC2655106

[18] Zhang Y, Lou B, Lal RB, Gettie A, Marx PA, et al (2000) Use of inhibitors to evaluate coreceptor usage by simian and simian/human immunodeficiency viruses and human immunodeficiency virus type 2 in primary cells. J Virol 74: 6893–6910. 10888629 10.1128/jvi.74.15.6893-6910.2000 PMC112207

